# Malignant Progression Contributes to the Failure of Combination Therapy for Atypical Meningiomas

**DOI:** 10.3389/fonc.2020.608175

**Published:** 2021-01-15

**Authors:** Qing Zhang, Zheng Wen, Ming Ni, Da Li, Ke Wang, Gui-Jun Jia, Zhen Wu, Li-Wei Zhang, Wang Jia, Liang Wang, Jun-Ting Zhang

**Affiliations:** ^1^Department of Neurosurgery, Chinese People’s Liberation Army General Hospital, Beijing, China; ^2^Department of Neurosurgery, Beijing Tian Tan Hospital, Capital Medical University, Beijing, China; ^3^China National Clinical Research Center for Neurological Diseases, Beijing, China; ^4^Center of Brain Tumor, Beijing Institute for Brain Disorders, Beijing, China; ^5^Beijing Key Laboratory of Brain Tumor, Beijing, China

**Keywords:** malignant progression, Ki-67, atypical meningioma, gross-total resection, external beam radiotherapy

## Abstract

**Objective:**

To investigate the independent risk factors for recurrence in intracranial atypical meningiomas (AMs) treated with gross total resection (GTR) and early external beam radiotherapy (EBRT).

**Methods:**

Clinical, radiological, and pathological data of intracranial AMs treated with GTR-plus-early-EBRT between January 2008 and July 2016 were reviewed. Immunohistochemical staining for Ki-67 was performed. Kaplan–Meier curves and univariate and multivariate Cox proportional hazards regression analyses were used to explore independent predictors of tumor recurrence. Chi square test was performed to compare variables between subgroups.

**Results:**

Forty-six patients with intracranial AMs underwent GTR and early EBRT. Ten (21.7%) recurred and three (6.5%) died during a median follow-up of 76.00 months. Univariate and multivariate Cox analyses revealed that malignant progression (MP) (P = 0.009) was the only independent predictor for recurrence, while Ki-67 was of minor value in this aspect (P = 0.362). MP-AMs had a significantly higher recurrence rate (P = 0.008), a higher proportion of irregularly shaped tumors (P = 0.013) and significantly lower preoperative Karnofsky Performance Scale (KPS) scores (P = 0.040) than primary (Pri) AMs. No significant difference in Ki-67 expression was detected between these subgroups (P = 0.713).

**Conclusions:**

MP was significantly correlated with an increased incidence of recurrence in GTR-plus-early-EBRT-treated intracranial AMs. Significantly higher frequencies of tumor relapse and irregularly shaped tumors and lower preoperative KPS scores were observed in MP-AMs compared with Pri-AMs. Ki-67 expression is of minor value in predicting tumor recurrence or distinguishing tumor origins in AMs.

## Introduction

Meningiomas are the most common primary intracranial tumors (1). Among the three World Health Organization (WHO) grades of meningiomas, WHO grade II meningiomas are further classified into three subtypes: AMs, chordoid meningiomas, and clear cell meningiomas (2). Their reported incidence increased as the WHO classification was updated, ranging from 19 to 35.5% of all meningiomas in the literature (3–5). They exhibit a higher recurrence rate (up to 30%) and an unfavorable survival outcome than benign meningiomas (BMs, WHO grade I) (4). Treatment approaches for malignant meningiomas (MMs, WHO grade III) were referenced to improve this unsatisfactory prognosis (6). Surgical resection is the primary treatment, and the extent of resection is considered the most important factor for predicting recurrence and survival (7, 8). Previous studies have also demonstrated that adjuvant radiotherapy significantly improves progression-free survival (PFS) and overall survival (OS) after subtotal resection (STR) of AMs (9). However, its efficiency in those following GTR remains heavily debated (10) and has consequently led to non-uniform clinical decision-making across institutions (6). Confounding effects of different subtypes of WHO grade II meningiomas (11–14), different radiation methods (15, 16), timing of radiation (17–19), *etc*., in previous studies may have contributed to this uncertainty and complicated the exploration of possible prognostic factors. Therefore, these effects were eliminated in the present study to target the precise reasons for the recurrence of GTR-plus-early-EBRT-treated intracranial AMs.

## Materials and Methods

### Inclusion Criteria and Clinical Data Collection

Medical records and radiologic data of intracranial AM patients who underwent operations in the Department of Neurosurgery, Beijing Tian Tan Hospital, Capital Medical University from January 2008 to July 2016 were reviewed. All pathology slides were centrally reviewed and graded based on the 2016 revision of the WHO classification of tumors of the central nervous system (20) (independently by two neuropathologists blinded to clinical history, and a senior neuropathologist made the judgment if there was a discrepancy). Patients who underwent GTR as well as adjuvant EBRT at their initial pathological diagnosis of AM were included. The following exclusion criteria were adopted to explore prognostic factors more objectively: 1) pathological diagnosis of chordoid or clear cell meningioma; 2) received any other form of radiotherapy; 3) without explicit documentation of an EBRT plan; 4) lack of timely adjuvant EBRT [which was defined as within 6 months postoperatively in the literature (17)] or EBRT was postponed/terminated early; and 5) diagnosis of neurofibromatosis.

Data of the included patients were compiled from medical records, imaging, and pathological tests, and other records provided by the patients themselves. Follow-ups were performed by postoperative outpatient visits. The extent of resection was based on both the surgeon’s impression during surgery and our review of the first postoperative magnetic resonance imaging (MRI) scans. GTR was defined as Simpson grades I–II. AMs were stratified into the Pri group and the MP group based on tumor origins. MP-AMs refer to AMs who were pathologically diagnosed as BMs in previous surgeries and/or histopathologically confirmed to transform into MMs in subsequent surgeries. Others without any documentation of progression were considered as Pri-AMs. Tumor location was divided into the skull base group (including sphenoidal ridge, petroclival, foramen magnum, middle fossa, olfactory groove and orbital meningiomas) and the non-skull base group (including convexity, parasagittal, falx, cerebellar convexity, lateral ventricular and tentorial meningiomas). Tumor shape was classified as either irregular or regular based on the presence or absence of lobulation at the tumor–brain interface (mushroom-shaped tumors were included in the irregularly shaped group). PFS was defined as the period between the onset of surgery prior to EBRT and the observation of imaging-verified disease progression. OS was defined as the period from the date of surgery prior to EBRT to death or the last follow-up.

### Pathological Examination

All AM samples were obtained during the surgery right before EBRT and were formalin-fixed and paraffin-embedded (FFPE) postoperatively. Hematoxylin and eosin (HE) staining and immunohistochemical staining for Ki-67 (the primary Ki-67 antibody was obtained from Abcam, Cambridge, Massachusetts, USA) were performed.

### Statistical Analysis

The baseline patient characteristics are summarized as percentages for categorical variables and as the mean ± standard deviation for continuous variables. Univariate and multivariate Cox proportional hazards regression analyses were used to assess correlations between various factors and recurrence. Hazard ratios (HRs) with 95% confidence intervals (CIs) were calculated. Kaplan–Meier curves were generated to graphically display the associations between variables and PFS. Chi square test was performed to compare variables between different subgroups. All P values are two-sided, and significance was defined using a threshold of 0.05. Statistical analyses were performed with SPSS Statistics software (version 19.0; IBM Corporation, Armonk, NY, USA). The hospital ethics committee approved this study, and all patients provided written consent.

## Results

### Patient Demographics and Tumor Characteristics

A total of 46 intracranial AM patients met the aforementioned criteria, including 25 (54.3%) males and 21 (45.7%) females. The male-to-female ratio was 1.19:1. The mean age at the first presentation of AM was 49.67 ± 13.15 years (range, 20–77 years). The median surgery-radiation interval was six weeks (range, 2–21 weeks). The median radiation dose of EBRT was 60 Gy (range, 50–63 Gy; delivered to the tumor bed in 1.8- to 2.0-Gy fractions). Ten patients (21.7%) experienced tumor relapse and three patients (6.5%) died before the last follow-up (May 2020). All these three fatalities were due to meningiomas. The median follow-up duration was 76.00 months (range, 48–144 months). The median PFS was 73.50 months (range, 21–144 months). 16 (34.8%) AMs experienced MP. All of these 16 patients progressed from BMs before the combination therapy, and one of them experienced another transformation (from AM to MM) during the follow-up (**Table 1**).

**Table 1:** Baseline characteristics of 46 combination-therapy-treated intracranial AM patients.

Characteristic	Total (n = 46)	Pri-AM (n = 30)	MP-AM (n = 16)
Tumor origin, n (%)	46 (100.0)	30 (65.2)	16 (34.8)
Age (years)			
Median (range)	53 (20–77)	53.50 (24–77)	47 (20–65)
Mean ± SD	49.67 ± 13.15	51.13 ± 12.58	46.94 ± 14.17
Gender, n (%)			
Female	21 (45.7)	13 (43.3)	8 (50.0)
Male	25 (54.3)	17 (56.7)	8 (50.0)
Tumor location, n (%)			
Non-skull base	33 (71.7)	22 (73.3)	11 (68.8)
Convexity	11	7	4
Parasagittal	5	3	2
Falx	7	6	1
Cerebellar convexity	3	2	1
Lateral ventricular	4	1	3
Tentorial	3	3	0
Skull-base	13 (28.3)	8 (26.7)	5 (31.3)
Sphenoidal ridge	5	3	2
Petroclival	4	2	2
Foramen magnum	1	1	0
Middle fossa	1	1	0
Olfactory groove	1	1	0
Orbital	1	0	1
Max tumor diameter (mm)			
Median (range)	50 (12–100)	50.50 (24–79)	45.50 (12–100)
Mean ± SD	51.15 ± 18.02	52.20 ± 15.48	49.19 ± 22.46
Preoperative KPS			
Median (range)	80 (60–100)	90 (60–100)	80 (60–90)
Mean ± SD	82.61 ± 8.80	84.33 ± 8.58	79.38 ± 8.54
Postoperative KPS			
Median (range)	90 (70–100)	90 (70–100)	80 (70–90)
Mean ± SD	85.43 ± 7.52	86.33 ± 8.09	83.75 ± 6.19
Intraoperative blood loss (ml)			
Median (range)	400 (100–4,000)	400 (100–4,000)	350 (200–1,000)
Mean ± SD	638.04 ± 723.43	755.00 ± 859.57	418.75 ± 250.92
GTR + early EBRT, n (%)	46 (100.0)	30 (100.0)	16 (100.0)
Simpson grading, n (%)			
Grade I	37(80.4)	26 (86.7)	11 (68.8)
Grade II	9(19.6)	4 (13.3)	5 (31.3)
Surgery-radiation interval (weeks)			
Median (range)	6 (2–21)	5.50 (2–21)	6 (2–15)
Mean ± SD	6.67 ± 4.08	6.40 ± 4.22	7.19 ± 3.89
Radiation dose (Gy)			
Median (range)	60 (50–63)	60 (50–63)	60 (50–63)
Mean ± SD	57.97 ± 4.09	58.13 ± 4.09	57.69 ± 4.22
PFS (months)			
Median (range)	73.50 (21–144)	81.00 (21–144)	63.00 (21–112)
Mean ± SD	76.02 ± 28.27	82.80 ± 26.80	63.31 ± 27.28
Follow-up (months)			
Median (range)	76.00 (48-144)	81.00 (54-144)	72.50 (48-112)
Mean ± SD	81.89 ± 22.75	84.73 ± 24.36	76.56 ± 18.97
Recurrence, n (%)	10 (21.7)	3 (10.0)	7 (43.8)
Frequency of recurrence			
Median (range)	0 (0-3)	0 (0-1)	0 (0-3)
Mean ± SD	0.26 ± 0.61	0.07 ± 0.25	0.63 ± 0.89
Frequency of operation before MP			
Median (range)	——	——	1 (1-3)
Mean ± SD	——	——	1.31 ± 0.60
Pathways of MP, n (%)			
Benign to Atypical	——	——	15 (93.8)
Benign to Atypical to malignant	——	——	1 (6.7)
Death, n (%)	3 (6.5)	0 (0.0)	3 (18.8)

AM, atypical meningioma; EBRT, external beam radiotherapy; GTR, gross total resection; KPS, Karnofsky performance score; MP, malignant progression; PFS, progression-free survival; Pri, primary; SD, standard deviation.

### Univariate and Multivariate Analyses Associated With Tumor Recurrence

**Figure 1:**
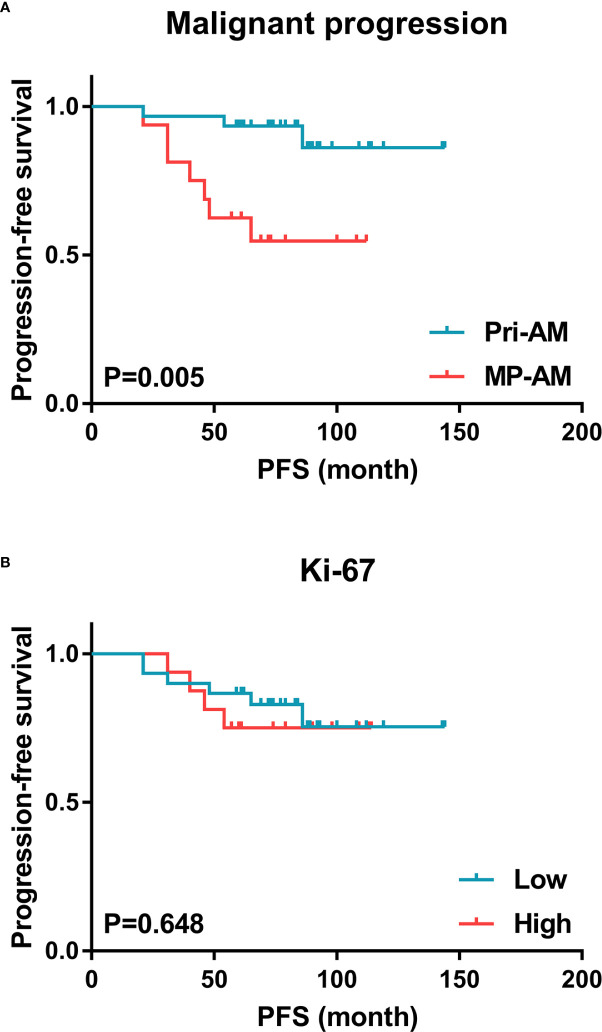
Kaplan–Meier estimates of PFS for combination-therapy-treated AM patients. **(A)** Malignant progression was a significant predictor of tumor recurrence in GTR-plus-early-EBRT-treated AMs, while **(B)** the Ki-67 expression level was of minor value in this respect (AM, atypical meningioma; PFS, progression-free survival).

The univariate Cox analysis showed that the PFS of GTR-plus-early-EBRT-treated AM patients was significantly influenced by MP (P = 0.012). A high radiation dose (≥60.0 Gy), a Simpson grade II resection and a skull base location were not significant prognostic factors for PFS. Since Ki-67 has been widely correlated with cell proliferation and the degree of malignancy of meningeal tumors (21, 22), both MP and Ki-67 were incorporated in the multivariate analysis. The multivariate Cox analysis revealed that only MP was an independent predictor of tumor recurrence in GTR-plus-early-EBRT-treated AMs (P = 0.009) (**Table 2**, **Figure 1**). Regarding OS, three patients died during the follow-up, all of whom experienced MP prior. However, this event number was too small to be used for further exploration of the prognostic factors of OS.

**Table 2:** Univariate and multivariable Cox regression predicting tumor recurrence in 46 combination-therapy-treated intracranial AM patients.

Variables	Univariate		Multivariate	
	HR(95%CI)	p	HR(95%CI)	p
Age ≥ 50 years	0.354(0.091–1.371)	0.133		
Male	0.844(0.244–2.926)	0.789		
MP-AM	5.676(1.454–22.167)	0.012*	6.354(1.571–25.697)	0.009*
Preoperative KPS ≥ 90	1.300(0.374–4.522)	0.679		
Postoperative KPS ≥ 90	0.870(0.245–3.086)	0.829		
Skull base group	1.109(0.286–4.304)	0.881		
Max tumor diameter ≥ 50.0 mm	0.848(0.245–2.933)	0.795		
Heterogeneous contrast enhancement	1.371(0.387–4.865)	0.625		
Cystic tumor	22.923(0.001–5.917 × 10^5^)	0.546		
Hemorrhage or necrosis	22.923(0.001–5.917 × 10^5^)	0.546		
Intratumoral calcification	21.143(0.000–3.650 × 10^8^)	0.720		
Nerves/vessels involved	1.628(0.420–6.320)	0.481		
Irregular-shaped	1.107(0.319–3.836)	0.873		
Ill-defined margins	1.523(0.323–7.187)	0.595		
Peritumoral edema	0.971(0.249–3.784)	0.967		
Midline shift	0.953(0.274–3.310)	0.940		
Compressed ventricles	0.474(0.060–3.745)	0.479		
Cerebral hernia	1.320(0.278–6.262)	0.727		
Empty sella	1.143(0.296–4.423)	0.846		
Larger intraoperative blood loss	2.221(0.570–8.648)	0.250		
Simpson grade II	1.070(0.227–5.040)	0.932		
Surgery-radiation interval ≥ 6 weeks	0.869(0.251–3.003)	0.824		
Radiation dose ≥ 60.0 Gy	0.583(0.164–2.069)	0.404		
Ki-67 ≥ 5%	1.340(0.377–4.771)	0.651	1.849(0.493–6.930)	0.362

AM, atypical meningioma; CI, confidence interval, HR, hazard ratio; KPS, Karnofsky performance score; MP, malignent progression.

*P value < 0.05 indicates statistical significance.

### Comparison Between Primary-Atypical Meningiomas and Malignant Progression-Atypical Meningiomas

The characteristics of Pri-AMs and MP-AMs were compared. The recurrence rate (P = 0.008) and the proportion of irregularly shaped tumors were significantly higher in MP-AMs than in Pri-AMs (P = 0.013), while the preoperative KPS score was significantly lower in MP-AMs than in Pri-AMs (P = 0.040). No significant differences in the radiation dose, surgery-radiation interval or Ki-67 expression level were detected between the groups (**Table 3**).

**Table 3:** Clinical characteristics of different origins of 46 combination-therapy-treated intracranial AMs.

Variable, n (%)	Overall	Tumor origin		
		Pri-AM	MP-AM	P
		(n = 30)	(n = 16)	
Age				0.292
<50 years	21(45.7)	12 (40.0)	9 (56.3)	
≥50 years	25(54.3)	18 (60.0)	7 (43.8)	
Gender				0.665
Female	21 (45.7)	13 (43.3)	8 (50.0)	
Male	25 (54.3)	17 (56.7)	8 (50.0)	
Preoperative KPS				0.040*
<90	25(54.3)	13 (43.3)	12 (75.0)	
≥90	21(45.7)	17 (56.7)	4 (25.0)	
Postoperative KPS				0.082
<90	18(39.1)	9 (30.0)	9 (56.3)	
≥90	28(60.9)	21 (70.0)	7 (43.8)	
Tumor location				1.000^†^
Non-skull-base	33 (71.7)	22 (73.3)	11 (68.8)	
Skull-base	13 (28.3)	8 (26.7)	5 (31.3)	
Max tumor diameter				0.292
<50.0 mm	21(45.7)	12(40.0)	9(56.3)	
≥50.0 mm	25(54.3)	18(60.0)	7(43.8)	
Contrast enhancement				0.421
Homogeneous	15(32.6)	11(36.7)	4(25.0)	
Heterogeneous	31(67.4)	19(63.3)	12(75.0)	
Cystic component				0.496^†^
Absent	43(93.5)	27(90.0)	16(100.0)	
Present	3(6.5)	3(10.0)	0(0.0)	
Hemorrhage or necrosis				1.000^†^
Absent	43(93.5)	28(93.3)	15(93.8)	
Present	3(6.5)	2(6.7)	1(6.3)	
Intratumoral calcification				1.000^†^
Absent	45(97.8)	29(96.7)	16(100.0)	
Present	1(2.2)	1(3.3)	0(0.0)	
Nerves/vessels involvement				0.869
Uninvolved	18 (39.1)	12 (40.0)	6 (37.5)	
Involved	28 (60.9)	18 (60.0)	10 (62.5)	
Shape of tumor				0.013*
Regular	23 (50.0)	19 (63.3)	4 (25.0)	
Irregular	23 (50.0)	11 (36.7)	12 (75.0)	
Tumor margins				0.635^†^
Well-defined	34 (73.9)	21 (70.0)	13 (81.3)	
Ill-defined	12 (26.1)	9 (30.0)	3 (18.8)	
Peritumoral edema				1.000^†^
Absent	14 (30.4)	9 (30.0)	5 (31.3)	
Present	32 (69.6)	21 (70.0)	11 (68.8)	
Midline shift				0.686
Absent	24 (52.2)	15 (50.0)	9 (56.3)	
Present	22 (47.8)	15 (50.0)	7 (43.8)	
Compressed ventricles				0.558^†^
Absent	8 (17.4)	4 (13.3)	4 (25.0)	
Present	38 (82.6)	26 (86.7)	12 (75.0)	
Cerebral hernia				0.813^†^
Absent	35 (76.1)	22 (73.3)	13 (81.3)	
Present	11 (23.9)	8 (26.7)	3 (18.8)	
Empty sella				0.066
Absent	31 (67.4)	23 (76.7)	8 (50.0)	
Present	15 (32.6)	7 (23.3)	8 (50.0)	
Intraoperative blood loss				0.829
<400 ml	22 (47.8)	14 (46.7)	8 (50.0)	
≥400 ml	24 (52.2)	16 (53.3)	8 (50.0)	
Simpson grading				0.285^†^
Grade I	37(80.4)	26 (86.7)	11 (68.8)	
Grade II	9(19.6)	4 (13.3)	5 (31.3)	
Surgery-radiation interval				0.418
<6 weeks	21 (45.7)	15 (50.0)	6 (37.5)	
≥6 weeks	25 (54.3)	15 (50.0)	10 (62.5)	
Radiation dose				1.000^†^
<60.0 Gy	14 (30.4)	9 (30.0)	5 (31.3)	
≥60.0 Gy	32 (69.6)	21 (70.0)	11 (68.8)	
Ki-67				0.713
<5%	30(65.2)	19 (63.3)	11 (68.8)	
≥5%	16(34.8)	11 (36.7)	5 (31.3)	
Recurrence				0.008*
Absent	36(78.3)	27 (90.0)	9 (56.3)	
Present	10(21.7)	3 (10.0)	7 (43.8)	

AM, atypical meningioma; KPS, Karnofsky performance score; MP, malignant progression; Pri, primary.

*P value <0.05 indicates statistical significance.

^†^Correction for continuity.

## Discussion

In the present study, MP was the only independent risk factor for tumor recurrence in GTR-plus-early-EBRT-treated AMs (**Table 2**). MP-AMs accounted for 34.8% (16/46) of the current series (**Table 1**). Except for their higher tendency of recurrence as compared with Pri-AMs, their lower preoperative KPS score and higher proportion of irregular-shaped tumors were also presented (**Table 3**). In addition, three patients died during follow-up, all of whom were MP-AM patients (**Table 1**).

### Malignant Progression Meningiomas

#### High Proportions of Malignant Progression in Recurrent Meningiomas and Non-Benign Meningiomas

The clinical value of MP has been underestimated due to its low incidence in the entire meningiomas (0.16 to 2%) (23, 24). However, MP-meningiomas account for a large proportion of recurrent meningiomas and non-benign meningiomas. 14 to 28.5% of recurrent BMs transform into atypical or malignant lesions (25–27), and this rate rises to approximately 26 to 33% in recurrent AMs (25, 27, 28). MP-meningiomas have been reported as high a proportion as 38% of AMs and 70% of MMs (29). In the present cohort, 34.8% (16/46) of AMs progressed from BMs (**Table 1**), consistent with previous literature; 43.8% (7/16) of MP-AMs recurred, which was significantly higher than that of Pri-AMs (10%, 3/30) (p = 0.008) (**Table 3**); and among these recurrent AMs, MP-AMs accounted for up to 70% (7/10). Due to our strict criteria, the current high proportions of MP-AMs failed to reflect the situations when GTR and/or early EBRT were not achieved. Nevertheless, these high frequencies of MP-meningiomas in recurrent meningiomas and non-benign meningiomas reflect the poor efficacy of the existing therapies on MP-meningiomas. Therefore, MP of meningiomas is of value and should be considered in the prognostic analyses.

### Unsatisfactory Therapeutical Efficacy in Malignant Progression Meningiomas

A prognostic benefit associated with Pri-meningiomas has been previously reported in the literature. Krayenbühl et al. demonstrated a statistically significant decrease in the survival time of MP-AMs (average, 1.95 years; range, 1.02–15.95 years) as compared with Pri-AMs (average, 5.36 years; range, 0.07–7.71 years), and they postulated that this difference was caused by the increased technical difficulty of GTR in reoperations and the more aggressive behavior of MP-AMs (29). Moliterno et al. exhibited an OS advantage in patients with Pri-MMs independent of the extent of resection (medium OS: Pri-MM, 3.0 years; MP-MM, 2.4 years), though this finding was prohibited from reaching statistical significance in their multivariate analysis by their small sample size (30). An OS disadvantage in MP-AM patients can also be observed in the present study since three patients died during follow-up and they were all MP-AM patients. Likewise, further analyses were also limited by the small event number, which may be due to the strict criteria applied. Meanwhile, MP was a significant independent predictive factor for tumor recurrence in combination-therapy-treated AMs (**Table 2**). These findings underscore the value and advantages of exploring an effective identification method of MP-meningiomas.

Even after administration of the combination therapy described herein, the recurrence risk of MP-AMs was still high (43.8%), which may question the necessity of adjuvant radiotherapy (**Table 1**). It has been demonstrated that ionizing radiation (IR) can enhance cellular invasion and induce malignant transformation in several cancer cells (including breast, lung, and liver cancer and glioma cells) (31–36). Our previous study confirmed that the invasiveness of IOMM-Lee meningioma cells can also be promoted by IR (37). In the context of the unsatisfactory efficacy of combination therapy in MP-AMs and the shortage of effective IR-induced MP-meningioma models (38), whether radiotherapy improves the prognosis of MP-AMs or stimulates them to undergo MP and recur requires further investigation.

#### Identification of Malignant Progression Meningiomas

At present, the clinical method of identifying MP meningiomas is based on the comparison between former and present pathologic diagnoses. However, for initial treatment, the effectiveness of this method is restricted. Continuous efforts have been made to identify MP-meningiomas cytogenetically and clinically. Accumulated evidences indicated that meningiomas can be classified into two distinct subtypes based on their origins: Pri- and MP-meningiomas (29, 30, 39). Meningiomas with different progression statuses possess variant molecular bases and display distinct clinical characteristics and behaviors (29, 30).

#### ① Cytogenetical Differences Between Primary- and Malignant Progression Meningiomas

A stepwise clonal evolution model was initially used to explain the MP in meningiomas (40), which states that the malignancy of meningiomas progresses as genetic alterations accumulate (41–44). That is, more aggressive meningiomas tend to present with more complex karyotypes (41). However, this model was proposed based on cytogenetic alterations in large groups of patients with different grades of tumors (39). It is more of a reflection of the difference between WHO grades than a reflection of the difference between prior- and post-status of MP. Moreover, complex karyotypes have been detected in BMs by Perry et al. (45). Based on an analysis of the biological and genetic findings in specimens of successive histological grades of each MP meningioma, a predetermined-progression notion was developed by Al-Mefty and his colleagues (39). They documented that the presence of complex karyotypes in benign tumors preceded the histopathological manifestation of malignancy, which raised the possibility that these tumors were intrinsically malignant and destined to progress. The clonal evolution model states that lower-grade tumors possess lower karyotype complexity, while the predetermined-progression notion states that complex karyotypes already exist in lower-grade statuses of MP-meningiomas. Hence, there is a possibility that, in meningiomas of a same grade, those with higher karyotype complexity may indicate that they are MP-meningiomas, otherwise they are may be Pri-meningiomas. As the only cytogenetic comparison of Pri- and MP-meningiomas to date, Krayenbühl et al. described higher frequencies of combined cytogenetic changes (chromosomes 1, 14 and 22) and monosomy of chromosomes 10 and 18 in MP-AMs and MP-MMs than in their Pri counterparts, respectively (29). Therefore, the Pri- and MP-meningiomas of a same grade may be distinguished by their karyotype differences.

#### ② Clinical Differences Between Primary- and Malignant Progression Meningiomas

The distribution of locations of Pri- and MP-meningiomas has been reported diversely. Based on a research with a high percentage of skull base meningiomas (61.1%, 22/36), Krayenbühl et al. reported that primary grade II-III meningiomas were predominately located in the cranial base (73.7%, 14/19), whereas progressed grade II–III meningiomas displayed a similar distribution in the skull base (47.1%) and non-skull base (52.9%) regions (29). In Moliterno’s study of MMs, the majority of tumors were located along the convexity/parasagittal areas (73.0%, 27/37). In their study, the majority of MP-MMs were located in the skull-base/posterior fossa (57%, 8/14), while Pri-MMs were discovered almost exclusively in the convexity/parasagittal regions (91%, 21/23) (30). In the present study, in which non-skull base AMs accounted for 71.7% of the cohort, a non-skull base predominance in AMs was observed regardless of the progression status (Pri-AMs: 73.3%; MP-AMs: 68.8%) (**Table 3**).

In addition, Moliterno et al. also detected a slight female predominance in Pri-MMs, and all of the Pri-MMs with metastatic lesions in their series were located along the convexity/parasagittal area (30). In the present study, patients with MP-AMs had lower preoperative KPS scores than those with Pri-AMs, which might be due to their higher frequency of previous surgeries, and the tumors were more likely to be irregular-shaped, which might be attributed to differences in the growth velocity of different regions of the tumor (46) (**Table 3**).

### Minor Value of Ki-67 in the Recurrence Prediction and Origin Identification of Atypical Meningomas

#### Minor Value of Ki-67 in Predicting Recurrence of Gross Total Resection-Plus-Early-External Beam Radiotherapy-Treated Atypical Meningiomas

Ki-67 has been widely used in studies of the proliferative potential of meningiomas (22). A recent meta-analysis by Liu et al. indicated a significant adverse prognostic value of a high Ki-67 expression level in the prognosis of meningiomas, and 4% was recommended as the appropriate cutoff value (47). Of their 43 included studies (comprising 5012 patients), only seven specifically targeted WHO grade II meningiomas and evaluated the prognostic value of Ki-67 expression in tumor recurrence (11–14, 48–50). Each of these seven studies met at least two of the following situations: 1) inclusion of chordoid and/or clear cell meningiomas; 2) with/without postoperative radiotherapy and/or different radiotherapy modalities; and 3) diverse Ki-67 cutoff values. Based on a relatively short follow-up (1–50 months; median: 10 months), Siegers et al. stated that differences in Ki-67 expression could not be observed between three recurring and 49 non-recurring meningiomas (51). Defining non-recurring meningiomas as those without recurrence at least 8 years postoperatively, Maj-Lis Møller and Otto Brændstrup detected no significant differences in the Ki-67 labeling index between recurring and non-recurring meningiomas, when either totally and subtotally resected tumors were studied or when only radically resected tumors were studied (52). Likewise, our present results suggest that the Ki-67 expression level cannot be used as a predictor of recurrence in GTR-plus-early-adjuvant-EBRT-treated AMs. The possible reasons may be as follows. First, tumor recurrence is not dependent solely on the proliferative status of cells, especially for tumors that have undergone radical GTR. Second, the mitotic index, a proliferation marker, has been utilized as a standard in the WHO classification of meningiomas (49, 53). Therefore, the difference in tumor cell proliferation ability among meningiomas of the same grade is not as obvious as that among meningiomas of different grades. The expression of Ki-67, another proliferation marker, is also associated with cell proliferation (54). Its labeling index determines the growth fraction of tumors in percentages and is widely used to estimate tumor prognoses. The Ki-67 expression level fluctuates throughout the cell cycle, peaks in mitosis (M phase) but is absent in the resting phase (G_0_ phase) (55, 56). Consequently, the correlation between the peak expression level of Ki-67 in mitosis and the mitotic index leads to a minor difference in Ki-67 expression among meningiomas of the same WHO grade. Third, the abovementioned differential expression of Ki-67 among phases is also related to its role in estimating radioresistance (49). It has been substantiated that meningiomas with a higher Ki-67 labeling index may be more susceptible to adjuvant radiotherapy (49). In the present study, all the samples were obtained before IR, and all the patients received EBRT postoperatively. Hence, it is possible that some of these AMs with higher Ki-67 expression might present higher radiosensitivity to EBRT and obtain better prognoses thereafter. To a certain extent, these aforementioned points might restrict Ki-67’s ability to predict tumor recurrence in GTR-plus-early-adjuvant-EBRT-treated AMs.

#### Minor Value of Ki-67 in the Origin Identification of Atypical Meningiomas

The positive correlation between the Ki-67 expression level and the degree of malignancy of meningeal tumors has also been reported (21), yet this conclusion was derived mostly from studies including multiple grades of meningiomas. In a study of meningiomas with the same WHO grade yet different origins, Krayenbühl and colleagues explored a statistically significant increase in the number of MP-AM patients with high proliferative indices (a Ki-67 index greater than 5% was considered high) compared with Pri-AM patients. However, it should be noted that only 20 patient samples were stained for Ki-67. Maj-Lis Møller and Otto Brændstrup detected no differences between the Ki-67 labeling index of BMs that recurred as BMs, WHO grade II meningiomas or MMs. In other words, the expression level of Ki-67 cannot be used to judge whether a BM will experience MP. Similarly, it cannot be used to determine whether an AM is primary or malignant progressed based on our results (**Table 3**). According to Al-Mefty’s theory, some lower-grade meningiomas that harbor complex genetic aberrations are predetermined to histopathological progression to malignancy. They also stated that proliferation indices denoted something that was already occurring in the tumor cells more than they predicted the tumor’s potential behavior. That is to say, MP in meningiomas is a predestined but gradually manifested process. The proliferation index at one certain point in time cannot fully reflect the pre- or post-MP state of these cells. This may explain the current inability to determine the genesis of AMs by the expression level of Ki-67.

## Limitations

Potential limitations of this study should be taken into consideration. First, selection bias is inevitable due to the single-center-based retrospective design and the selection of GTR-plus-early-EBRT-treated AMs as the research object. Second, the present rigorous criteria restricted the sample size and the statistical power, and the small event number of death further restricted the exploration of the prognostic factors for OS in GTR-plus-early-EBRT-treated AMs and its difference between Pri-AMs and MP-AMs. Third, the present identification method of MP in meningiomas was based on the comparison between former and present pathologic diagnoses. Hence, there still exist uncertainties that some Pri-AMs in the present study may arise from BMs before any surgery or progress to MMs in the future even though the shortest follow-up period in the current cohort exceeded the reported mean period for MP-AM progression to MM (39.8 months) (57).

## Conclusions

MP is the only independent predictor of tumor recurrence in GTR-plus-early-EBRT-treated AMs. Satisfactory efficacy was not achieved in MP-AMs even after radical combination therapy. Significant higher frequencies of tumor relapse and irregularly shaped tumors as well as lower preoperative KPS scores were observed in MP-AMs than in Pri-AMs. The Ki-67 expression level is of minor value in predicting tumor recurrence or distinguishing tumor origins in AMs. More accurate and effective methods to distinguish MP-AMs from Pri-AMs are required. Further comparisons between MP-AMs with or without adjuvant radiotherapy after GTR, and the construction of effective IR-induced MP-meningioma models will be helpful to assess the necessity of radiotherapy in preventing the recurrence of MP-AMs.

## Data Availability Statement

The original contributions presented in the study are included in the article/supplementary materials; further inquiries can be directed to the corresponding authors.

## Ethics Statement

The studies involving human participants were reviewed and approved by the ethics committee of Beijing Tian Tan Hospital. The patients/participants provided their written informed consent to participate in this study.

## Author Contributions

All authors conceptualized and made the experimental design. QZ, ZWe, MN, DL, and KW acquired the data. QZ, ZWe, MN, DL, and KW analyzed and interpreted the data. QZ drafted the article. All authors critically revised the article. WJ, JZ, and LW provided administrative/technical/material support. All authors contributed to the article and approved the submitted version.

## Funding

This work was supported by the National Natural Science Foundation of China (grant no. 81472370 to JZ).

## Conflict of Interest

The authors declare that the research was conducted in the absence of any commercial or financial relationships that could be construed as a potential conflict of interest.

## Abbreviations

AM, atypical meningioma; BM, benign meningioma; CI, confidence interval; EBRT, external beam radiotherapy; FFPE, formalin-fixed and paraffin-embedded; GTR, gross total resection; HE, hematoxylin and eosin; HR, hazard ratio; KPS, Karnofsky Performance Scale; MM, malignant meningioma; MP, malignant progression; MRI, magnetic resonance imaging; OS, overall survival; PFS, progression-free survival; Pri, primary; STR, subtotal resection; IR, ionizing radiation.
